# Preparation of Continuous Alumina Fiber with Nano Grains by the Addition of Iron Sol

**DOI:** 10.3390/ma13235442

**Published:** 2020-11-29

**Authors:** Luqun Liu, Juan Wang, Yunzhu Ma, Wensheng Liu, Shuwei Yao

**Affiliations:** National Key Laboratory of Science and Technology for National Defense on High-strength Structural Materials, Central South University, Changsha 410083, China; lluqun@csu.edu.cn (L.L.); wangjuan@csu.edu.cn (J.W.); zhuzipm@csu.edu.cn (Y.M.); liuwensheng@csu.edu.cn (W.L.)

**Keywords:** alumina fiber, iron sol, holding temperature, colloidal particle size, grain size

## Abstract

Continuous alumina fiber exhibits excellent mechanical properties owing to its dense microstructure with fine grains. In this study, alumina fiber was prepared by the sol–gel method using iron sol as a nucleating agent. It was proposed that the α-Al_2_O_3_ grain size be adjusted based on the modification of colloidal particle size. The effect of holding temperature and reaction material ratio on the iron colloidal particle size was studied. The microstructure of alumina fiber was characterized by scanning electron microscopy (SEM). The experiment results indicated that iron colloidal particle size increases with the holding temperature and the NH_4_HCO_3_/Fe(NO_3_)_3_·9H_2_O ratio. The alumina fiber with uniform nano α-Al_2_O_3_ grains was obtained by calcination at 1400 °C for 5 min. The mean grain size tends to rise with the mean colloidal particle size. Using the iron sol as a nucleating agent, the fiber with a mean grain size of 22.5 nm could be formed. The tensile strength of fibers increased with the decrease of grain size.

## 1. Introduction

Continuous alumina fiber has been widely applied in various fields such as aerospace, military industry and automobile, owing to its high strength, high modulus and exceptional oxidation resistance [1,2,3,4,5,6]. The excellent mechanical properties of alumina fiber mainly depend on the dense nanocrystalline structure. For example, Nextel^TM^ 610 fiber and FP fiber are typical alumina fibers with high purity of α-Al_2_O_3_ (>99 wt.%). The Nextel^TM^ 610 fiber, having a grain size of ~100 nm, exhibits high tensile strength of greater than 3.1 GPa [3], while the tensile strength of FP fiber is only about 1.4 GPa due to its larger grains (>500 nm) [5]. Hay et al. [6] found that the tensile strength of Nextel^TM^ 610 fiber was reduced with the growth of α-Al_2_O_3_ grains. Thus, controlling the grain size is essentially important in order to prepare high performance alumina fiber.

Generally, alumina fiber is prepared by the sol-gel method [7,8,9,10,11,12]. In this method, complex phase transformations of Al_2_O_3_ take place. The fiber transfers from amorphous to metastable phases (η, δ, γ, etc.) and then transforms into α-Al_2_O_3_. Since the transformation temperature of metastable phases to α-Al_2_O_3_ is about 1200 °C, it is challenging to avoid α-Al_2_O_3_ grain from coarsening during the transformation and densification processes. In order to obtain fiber with fine grains, nano α-Al_2_O_3_, α-Fe_2_O_3_ and α-Cr_2_O_3_ serving as seeds are added in alumina fiber, which induce the heterogeneous nucleation of α-Al_2_O_3_ at a lower temperature [13,14,15,16,17,18]. Yamamura et al. [14] found that the alumina gel added with 5 wt.% α-Al_2_O_3_ particles partially crystallized to α-Al_2_O_3_ by annealing at temperature as low as 600 °C. Li et al. [19] revealed that the addition of 3 wt.% α-Al_2_O_3_ seeds lowered the γ-to-α transformation temperature by 175 °C. As a result, the primary α-Al_2_O_3_ grain size decreased from 56 to 30 nm. Kumagai et al. [20] found that the introduction of nano α-Al_2_O_3_ seeds resulted in a dense microstructure with α-Al_2_O_3_ grain size of 100–400 nm at 1200 °C. In addition, Nextel^TM^ 610 fiber, the most representative alumina fiber, was also successfully produced by adding 0.7 wt.% Fe_2_O_3_. The addition of Fe_2_O_3_ provided the benefits of forming fine α-Al_2_O_3_ grains with the uniform size of 100 nm. In theory, increasing nucleation sites leads to the formation of more α-Al_2_O_3_ grains, subsequently contributing to refine grains. Li et al. [19] found that the grain size of the alumina fiber sintered at 1100 °C decreases from 86.7 to 74.3 nm, when the weight percent of α-Al_2_O_3_ seeds rises from 1% to 3%. However, the content of nucleating agent cannot be added indefinitely in order to obtain continuous high purity alumina fiber.

Theoretically, reducing the seed size is an effective way to increase the amount of nucleation sites when the content of the nucleating agent is constant. Xie et al. [21] compared the effect of α-Al_2_O_3_-seed size on the transformation from κ to α phase. Employing the same content of nucleating agent, the sample added with finer α-Al_2_O_3_ powder exhibits a smaller average particle size with a narrow distribution. However, the size of α-Al_2_O_3_, Fe_2_O_3_ and α-Cr_2_O_3_ powders is limited by the production technologies, which is usually lager than 10 nm. Considering the agglomeration of nanoparticles, the nucleation seed size is almost larger than the particle size of powders. Thus, employing powders as nucleating agents, the seed size is difficult to further decrease. Wilson et al. [22] prepared a dense α-Al_2_O_3_ fiber with finer grains (200–300 nm) through the addition of iron sol. It was confirmed that the iron sol could be transformed into α-Fe_2_O_3_ before the formation of α-Al_2_O_3_ and accelerating the transformation from metastable phases to α-Al_2_O_3_. Compared with powders, the size of colloidal particles can be controlled in the range of 1–100 nm. In addition, the iron colloidal particles can be uniformly distributed in the aluminum sol. However, these factors affecting iron colloidal particle size are unclear. The influence of iron colloidal particle sizes on the transformation of Al_2_O_3_ and the morphology of α-Al_2_O_3_ grains has not been reported.

In this paper, it was proposed to prepare alumina fiber with ultra-fine grains by adjusting the iron colloidal particle size. Fe(NO_3_)_3_·9H_2_O and NH_4_HCO_3_ were employed to prepare the iron sol. The effect of the material ratio and reaction temperature on the microstructure of iron colloidal particles was investigated. Alumina fiber with the nano grain size was prepared using iron sol as a nucleating agent. The relationship between the iron colloidal particle size and the grain size of α-Al_2_O_3_ fiber was discovered. The effect of iron sol on the microstructure of alumina fiber was discussed.

## 2. Materials and Methods

### 2.1. Iron Sol Preparation

Analytical pure Fe(NO_3_)_3_·9H_2_O and NH_4_HCO_3_ provided by Aladdin (Shanghai, China) were used to prepare the iron sol. Firstly, Fe(NO_3_)_3_·9H_2_O and NH_4_HCO_3_ were dissolved in a proper amount of water at room temperature. The weight ratios of Fe(NO_3_)_3_·9H_2_O/H_2_O and NH_4_HCO_3_/H_2_O were set to be at 1:23.5 and 1:10, respectively. Then, a certain amount of NH_4_HCO_3_ solution was slowly added (3 drops/s) into the rapidly stirred Fe(NO_3_)_3_ solution, which was placed in a 500 mL conical flask and stirred by a magnetic stirrer. The molar ratios of NH_4_HCO_3_ to Fe(NO_3_)_3_·9H_2_O were set to be at 1.5, 1.75, 2.0, 2.25 and 2.5. Finally, the mixed solution was held at a certain temperature for 1 h. The holding temperature varied from 25 to 80 °C.

### 2.2. Preparation of Alumina Fibers

In this study, alumina fiber was prepared by the sol-gel method, which includes the preparation of alumina precursor sol, concentration of the precursor sol and the spinning and sintering processes [23,24]. In this study, alumina powder (>99.5 wt.%, 1–3 μm), formic acid (99 wt.%), acetic acid (99.5 wt.%), nitric acid (65 wt.%) and deionized water (lab made) were employed to prepare the alumina precursor sol. The alumina powder, formic acid, acetic acid and nitric acid were all provided by Aladdin (Shanghai, China). The molar ratio of starting materials was set to be at 1:0.67:0.6:0.36:28. The alumina precursor sol was prepared at 85 °C in a single-layer glass reactor. The iron sol was added into the alumina precursor sol prior to the concentration process. The additional amount of iron sol was set in order to obtain a high-purity alumina fiber with 0.65 wt.% Fe_2_O_3_. The mixed solution was concentrated at 30–60 °C using a rotary evaporator (Shanghai Xiande Experimental Instrument Co., LTD, Shanghai, China) to obtain sols with a suitable viscosity (50–200 Pa∙s) for spinning. The precursor fiber was prepared using a lab-made dry spinning apparatus and collected by a bobbin winder. The precursor fiber was first preheated at 500 °C and then calcined at 1400 °C for 5 min using a tube furnace (BTF-1600C-IV-SL, BEQ, Hefei, China).

### 2.3. Characterization

The morphology of iron colloidal particles was observed by a high-resolution transmission electron microscopy (TEM, JEM-2100F, JEOL, Tokyo, Japan). The colloidal particle size and Zeta potential of iron sol were measured by a particle size analyzer (Zetasizer Nano ZS, Malvern, Malvern, England). The thermal decomposition behavior of iron sol was analyzed using a simultaneous thermal analyzer (STA-449C, Netzsch, Selb, Germany). The microstructure of calcined alumina fiber was characterized by a scanning electron microscopy (SEM, Nova Nano, FEI, Hillsboro, Oregon, USA). The fiber mean grain size was calculated by measuring the grain sizes using the image analysis software Images J (V1.51, National Institutes of Health, Bethesda, Maryland, America). More than 800 grains were randomly selected from the cross-section image and measured for each fiber. The tensile strength of the fiber was measured by a fiber strength tester (XS(08)XT-3, XuSai, Shanghai, China) at room temperatures. The gauge length of the tested fiber was set to be at 15 mm. The cross-sectional area of the fiber was measured by SEM. In this study, thirty samples were tested for each kind of fiber in order to acquire its strength values.

## 3. Results and Discussion

### 3.1. Effect of Preparation Conditions on the Size of Iron Colloidal Particles

**Figure 1:**
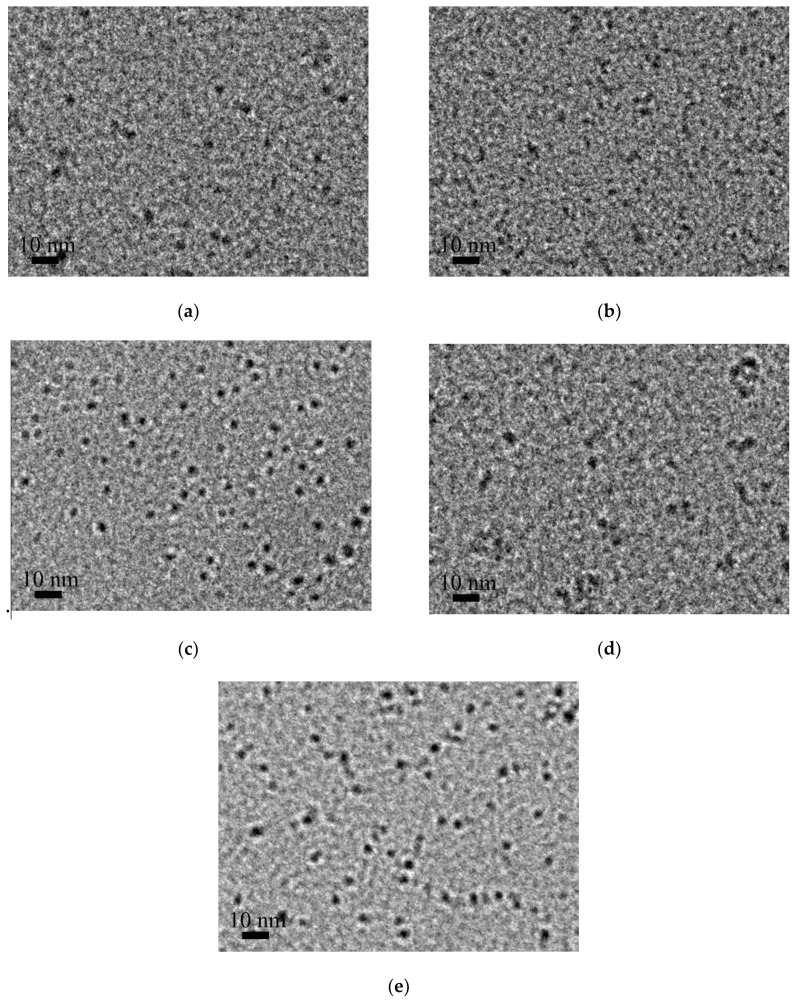
The morphology of iron colloidal particles prepared at different temperatures: (**a**) 40 °C; (**b**) 50 °C; (**c**) 60 °C; (**d**) 70 °C; (**e**) 80 °C.

In this study, iron sol was successfully prepared, which could be characterized by the color of dark red-brown. When a laser beam penetrated the sol, the Tyndall effect could be clearly observed. The morphology of iron colloidal particles obtained at different holding temperature is shown in Figure 1. In these cases, the molar ratio of NH_4_HCO_3_ to Fe(NO_3_)_3_·9H_2_O was set to be at 2.5. In Figure 1, the black dots correspond to iron colloidal particles. It can be seen that the colloidal particles are approximately spherical with a particle size of less than 10 nm. When the holding temperature rises, the colloidal particles tend to be larger.

**Figure 2:**
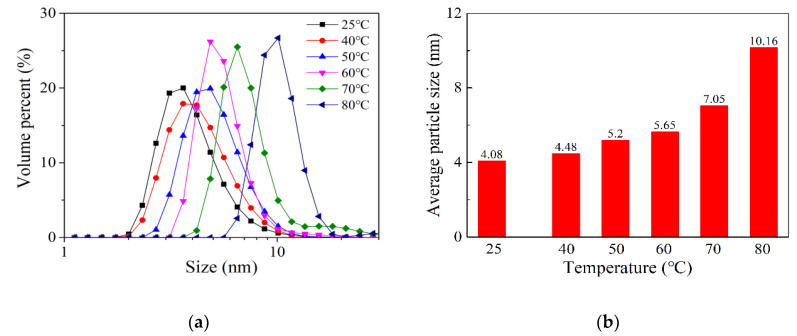
The size of iron colloidal particles prepared at different temperatures: (**a**) size distribution; (**b**) average size.

The colloidal particle size of iron sol was measured by a particle size analyzer. As shown in Figure 2a, the colloidal particle size follows the lognormal distribution. The sol that was prepared at temperatures ranging from 25 to 70 °C, its colloidal particle size mainly distributed in the range of 2–10 nm. With the increase of holding temperature, the distribution curve of colloidal particle size shifted to the right, which means that large colloidal particles tend to be formed at a higher temperature. When the holding temperature increased to 80 °C, the colloidal particle size varied from 5 to 20 nm, which is much larger than others. Figure 2b shows the average size of iron colloidal particles. It was also found that the average size sharply increases from 7.06 to 10.16 nm, when the holding temperature rose from 70 to 80 °C. Compared with the TEM results, the colloidal particle size obtained by the particle size analyzer is a little larger. This is because water in iron colloidal particles was lost during the preparation of the TEM sample. As a result, the size of iron colloidal particles decreased. Based on the above results, it is confirmed that increasing holding temperature contributes to larger iron colloidal particles being formed.

**Figure 3:**
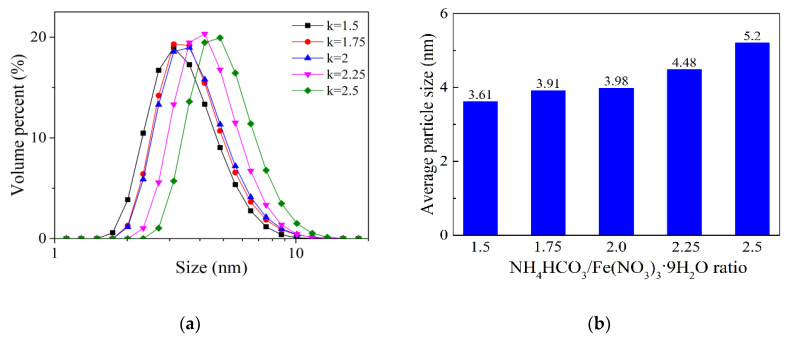
The size of iron colloidal particles prepared with different NH_4_HCO_3_/Fe(NO_3_)_3_·9H_2_O ratios at 50 °C: (**a**) size distribution; (**b**) average size. k in Figure 3a represents NH_4_HCO_3_/Fe(NO_3_)_3_·9H_2_O ratio.

Figure 3a presents the effect of NH_4_HCO_3_/Fe(NO_3_)_3_·9H_2_O ratio on the size distribution of the iron colloidal particles prepared at 50 °C. As shown in this figure, iron colloidal particle size mainly distributes in the range of 2–10 nm. The distribution curves of colloidal particle size are similar to the iron sols prepared under different conditions. When the ratio rises from 1.5 to 2.5, the distribution curve shifts to the right a little. Figure 3b shows the relationship between the average colloidal particle size and the NH_4_HCO_3_/Fe(NO_3_)_3_·9H_2_O molar ratio. It can be seen that the average size of iron colloidal particles is only 3.61 nm when the NH_4_HCO_3_/Fe(NO_3_)_3_·9H_2_O ratio is set to be at 1.5. With the increase of the material ratio, the average colloidal particle size slightly increases from 3.61 to 5.2 nm.

**Figure 4:**
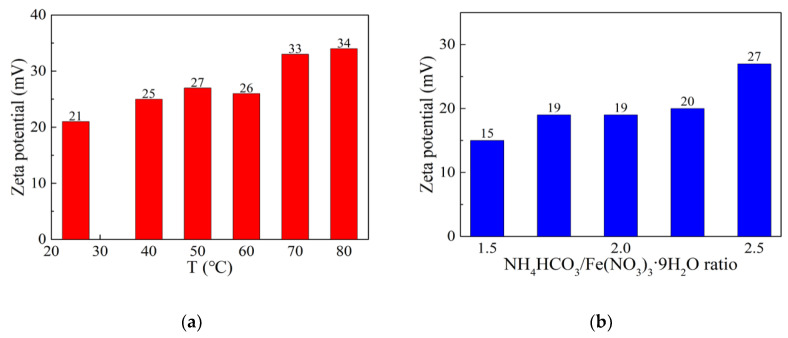
The Zeta potential of iron sols: (**a**) Zeta potential vs. holding temperature; (**b**) Zeta potential vs. NH_4_HCO_3_/Fe(NO_3_)_3_·9H_2_O ratio.

The stability of iron sol was studied by measuring the Zeta potential. As shown in Figure 4, the iron colloidal particles are positively charged, which is the same as alumina precursor colloidal particles. The zeta potential of iron sol ranges from 15 to 34 mV, indicating that all the sols are stable. Thus, the iron sol and alumina precursor sol can be mixed evenly without colloid coagulation. Among all sols, the one prepared at 50 °C with the NH_4_HCO_3_/Fe(NO_3_)_3_·9H_2_O ratio of 1.5 has the smallest zeta potential. This is probably related to its small colloidal particle size.

During the preparation process of iron sol, many complicated chemical reactions took place, such as the hydrolysis reaction and the polymerization reaction. It was discovered that hydrous iron polymers have the following formula [25],

[FeO_q_(OH)_x_(H_2_O)_p_^[3-(x + 2q)]+^]_n_[counterion^s−^]_[3_ − _(x + 2q)]n/s_ (1)

where s is the charge of the counterion having a value of 1, 2 or 3 and n can be larger than 500. The counterion can be any water-solubilizing anion, such as Cl^−^, NO^3−^ and COOH^−^. These complicated reactions can be simply described in the following equations:

(2) $${\mathrm{Fe}}^{3+}\;+\;H_{2}O\;⇌\;{\mathrm{Fe}(\mathrm{OH})}^{2+}\;+\;H^{+}$$

(3) $${\mathrm{Fe}(\mathrm{OH})}^{2+}\;+\;H_{2}O\;⇌\;{\mathrm{Fe}{\left(\mathrm{OH}\right)}_{2}}^{+}\;+\;H^{+}$$

(4) $$\mathrm{Fe}(\mathrm{OH}{{)}_{2}}^{+}\;+\;H_{2}O\;⇌\;\mathrm{Fe}(\mathrm{OH}{)}_{3}\;+\;H^{+}$$

(5) $${{\mathrm{NH}}_{4}}^{+}\;+\;H_{2}O\;⇌\;{\mathrm{NH}}_{4}(\mathrm{OH})\;+\;H^{+}$$

(6) $${{\mathrm{HCO}}_{3}}^{\text{–}}\;+\;H_{2}O\;⇌\;H_{2}{\mathrm{CO}}_{3}\;+\;{\mathrm{HO}}^{\text{–}}$$

(7) $${{\mathrm{HCO}}_{3}}^{\text{–}}\;+\;H^{+}\;⇌\;H_{2}O\;+\;{\mathrm{CO}}_{2}$$

(8) $${\mathrm{nFe}(\mathrm{OH}{)}_{z}}^{(3\;\text{–}\;z)+}\;+\;H_{2}O\;⇌\;[{\mathrm{FeO}}_{q}(\mathrm{OH}{)}_{x}{(H_{2}O{)}_{p}}^{[3-(x\;+\;2q)]+}{]}_{n}$$

In this study, adding more NH_4_HCO_3_ solution in the Fe(NO_3_)_3_ solution will consume more H^+^ and accelerate the hydrolysis reaction of Fe^3+^. Since the Fe^3+^ hydrolysis reaction is an endothermic reaction, the increasing temperature also promotes the hydrolysis reaction. As a result, the value of x increases with the increase of holding temperature or NH_4_HCO_3_/Fe(NO_3_)_3_·9H_2_O ratio. In the hydrous iron polymer, OH^–^ can work as a bridge joining the two Fe^3+^ together. When the value of x increases, the colloid particles become larger. Although the polymerization reaction is an exothermic reaction, a large energy barrier should be overcome during the polymerization process [26]. Thus, large colloidal particles tend to be formed at higher holding temperatures.

### 3.2. Thermal Analysis of Iron Sol

**Figure 5:**
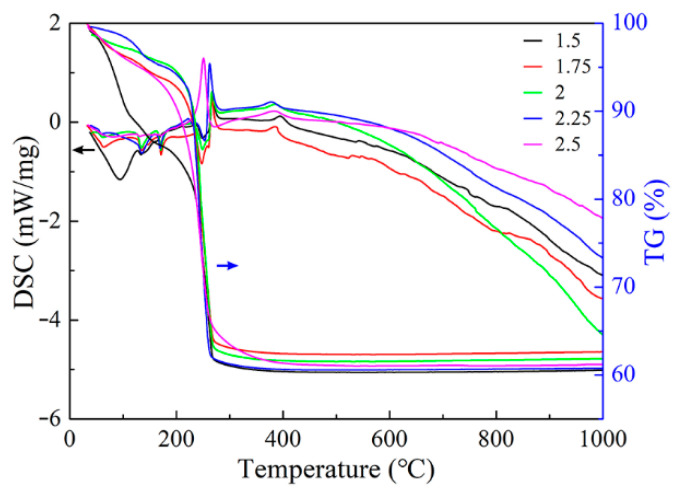
TG–DSC curves of the iron sol prepared with different NH_4_HCO_3_/Fe(NO_3_)_3_·9H_2_O ratio at 70 °C.

Figure 5 shows the TG–DSC curves of iron sols prepared with different NH_4_HCO_3_/Fe(NO_3_)_3_·9H_2_O ratios. It can be seen that these TG–DSC curves are similar for different samples. Thus, the iron sol prepared at 70 °C with the NH_4_HCO_3_/Fe(NO_3_)_3_·9H_2_O ratio of 2.5 was taken as an example to study its thermal decomposition behavior. These TG–DSC curves of iron sol obtained at a heating rate of 10 °C/min in air atmosphere are presented in Figure 5. The TG curve reveals that the mass loss of iron sol occurs from room temperature to about 400 °C, which can be divided into three stages. In the first stage, from room temperature to about 190 °C, the mass decreases slowly and the weight loss is about 7%. The second stage is from 190 °C to 270 °C. In this stage, the mass sharply declines from 93% to 64%. In the third stage (270–400 °C), the mass decreases gradually and the weight loss is only 3%. After that, the mass of iron sol keeps in constant. During the thermal decomposition process, the total weight loss is approximately 39%. As shown in Figure 5, there are three small endothermic peaks and two exothermic peaks in the DSC curve. All endothermic peaks distribute in the first stage. The two exothermic peaks are at 251 and 380 °C, respectively. Among all peaks, the one at 251 °C is the sharpest, which corresponds to the sharp decline in mass. According to the TG–DSC curves, it was inferred that the first stage of thermal decomposition is related to the removal of free water, bound water and ammonia gas. In the second stage, the dehydration and condensation of hydroxyl and the decomposition of Nitrate take place. In the third stage, the weight loss is probably caused by the removal of residual hydroxyl and Nitrate.

**Figure 6:**
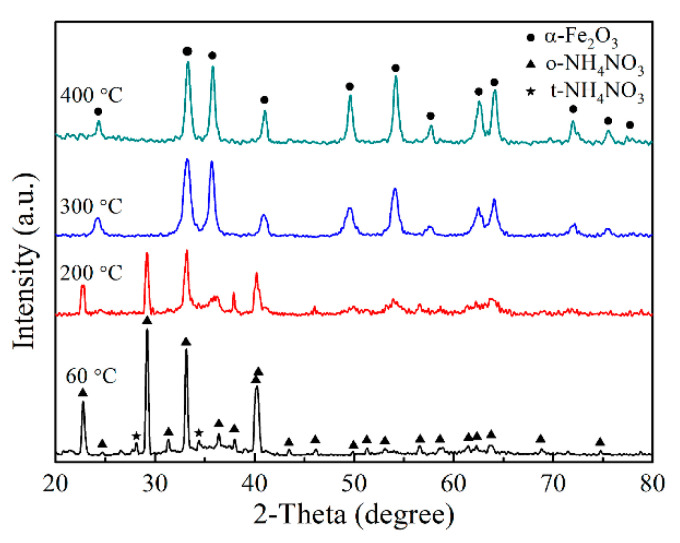
XRD patterns of the iron sol calcined at different temperatures for 1 h.

The exothermic peak at 380 °C is related to the transformation from iron sol to α-Fe_2_O_3_. It can be seen that α-Fe_2_O_3_ starts to form at about 350 °C. Figure 6 shows the XRD patterns of the iron sols. The iron sol dried at 60 °C for 12 h consists of orthorhombic NH_4_NO_3_ (o-NH_4_NO_3_), the amorphous phase and a small amount of tetragonal NH_4_NO_3_ (t-NH_4_NO_3_). The amorphous phase corresponds to the hydrous iron polymers. When the iron sol was annealed at 200 °C for 1 h, the thermal decomposition of iron sol occurred. As a result, the intensity of these peaks related to o-NH_4_NO_3_ and t-NH_4_NO_3_ decreases. When the sol was calcined at 300 or 400 °C for 1 h, the iron sol completely transformed into α-Fe_2_O_3_. Since the formation temperature of α-Fe_2_O_3_ is much lower than that of α-Al_2_O_3_, the iron sol can be served as a nucleating agent for alumina fiber.

### 3.3. The Effect of Iron Sol on the Microstructure of Calcined Alumina Fibers

In this study, the iron sols prepared with the NH_4_HCO_3_/Fe(NO_3_)_3_·9H_2_O ratio of 1.5, 2.0 and 2.5 at 50 °C and the sols obtained with the NH_4_HCO_3_/Fe(NO_3_)_3_·9H_2_O ratio of 2.5 at the temperature of 40, 50, 60, 70 and 80 °C were chosen as nucleating agents to be added in alumina fibers. For simplicity, the iron sol was labeled as T-*x* and K-*y*, where T represents the one being prepared at different temperatures with the NH_4_HCO_3_/Fe(NO_3_)_3_·9H_2_O ratio of 2.5, K corresponds to the sol obtained with different material ratio at 50 °C and x and y are the holding temperature and the NH_4_HCO_3_/Fe(NO_3_)_3_·9H_2_O ratio, respectively. The Al_2_O_3_ fibers prepared in this study are listed in Table 1.

**Figure 7:**
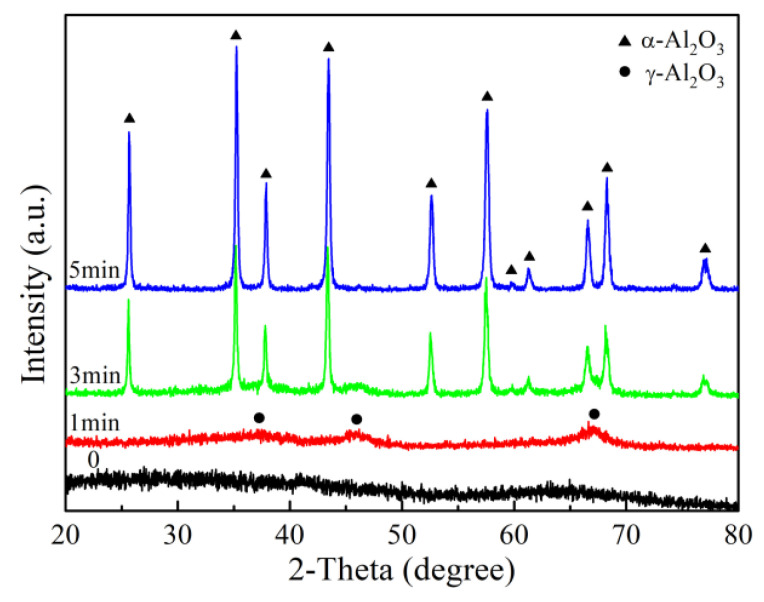
XRD patterns of Al_2_O_3_ fibers calcined at 1400 °C.

The Fe content in the calcined Al_2_O_3_ fibers was measured by inductively coupled plasma optical emission spectrometer (ICP-OES). The calculated Fe_2_O_3_ content based on the Fe content varied from 0.63% to 0.66%, which is in agreement with the setting value. The sample A4 was taken as an example to study the phase transformation of alumina fiber. Figure 7 shows the XRD patterns of this fiber. It can be seen that the amorphous phase exists only in the fiber preheated at 500 °C. When the fiber was calcined at 1400 °C for 1 min, the amorphous Al_2_O_3_ partially crystallized to γ-Al_2_O_3_. With the holding time prolonged, α-Al_2_O_3_ became the main phase. The fiber completely transformed into α-Al_2_O_3_ phase, when it was calcined for 5 min. Thus, all the alumina fibers were calcined at 1400 °C for 5 min.

**Figure 8:**
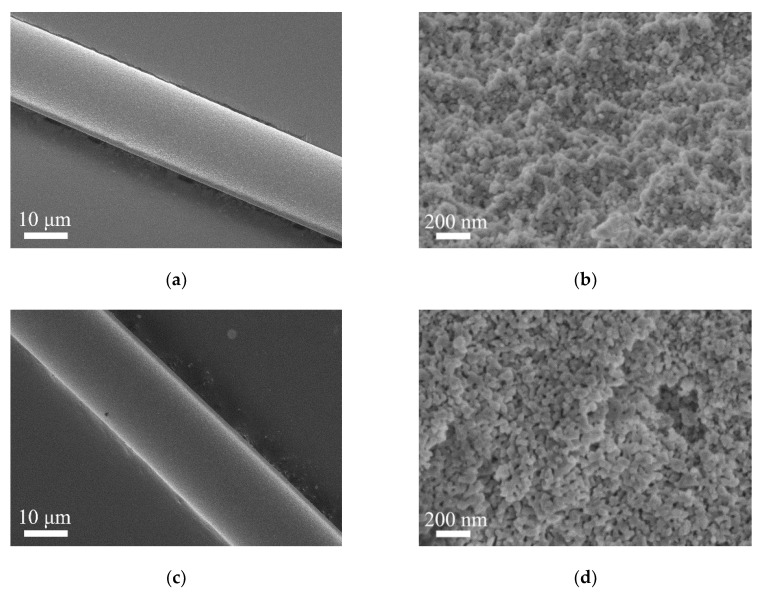

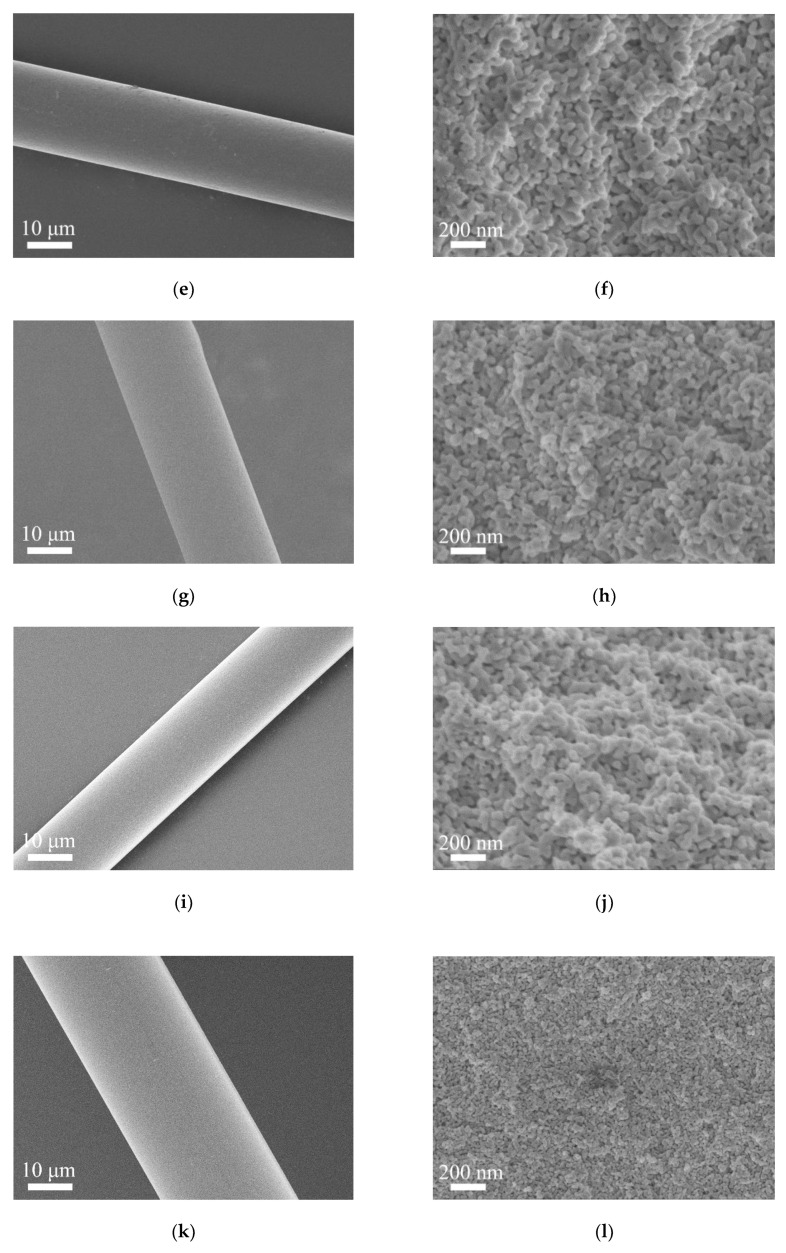

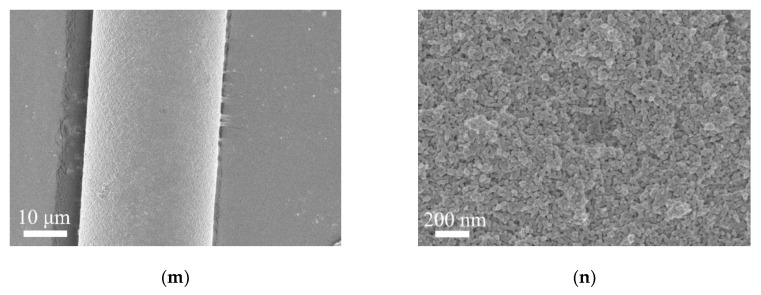
The morphology and cross-section microstructure of calcined Al_2_O_3_ fibers: (**a**,**b**) Sample-A1; (**c**,**d**) Sample-A2; (**e**,**f**) Sample-A3; (**g**,**h**) Sample-A4; (**i**,**j**) Sample-A5; (**k**,**l**) Sample-B1; (**m**,**n**) Sample-B2.

The morphology and cross-section microstructure of calcined Al_2_O_3_ fibers are shown in Figure 8. It can be seen that all the fibers are continuous with a diameter ranging from 15 to 18 µm. The fiber surface is smooth without obvious cracks, pores and other defects. The cross-section microstructure of alumina fiber is uniform and consists of nano-scale equiaxed grains. For different fibers, these microsturctures are similar except for their grain size. Among all fibers, the sample A1, B1 and B2 have a significantly smaller grain size. From sample A1 to A5, the α-Al_2_O_3_ grain size increases gradually.

**Figure 9:**
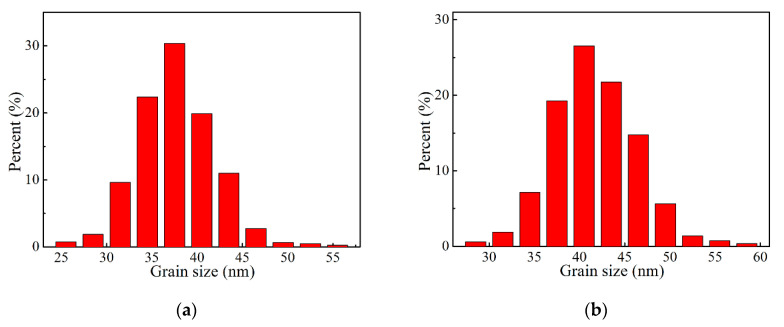

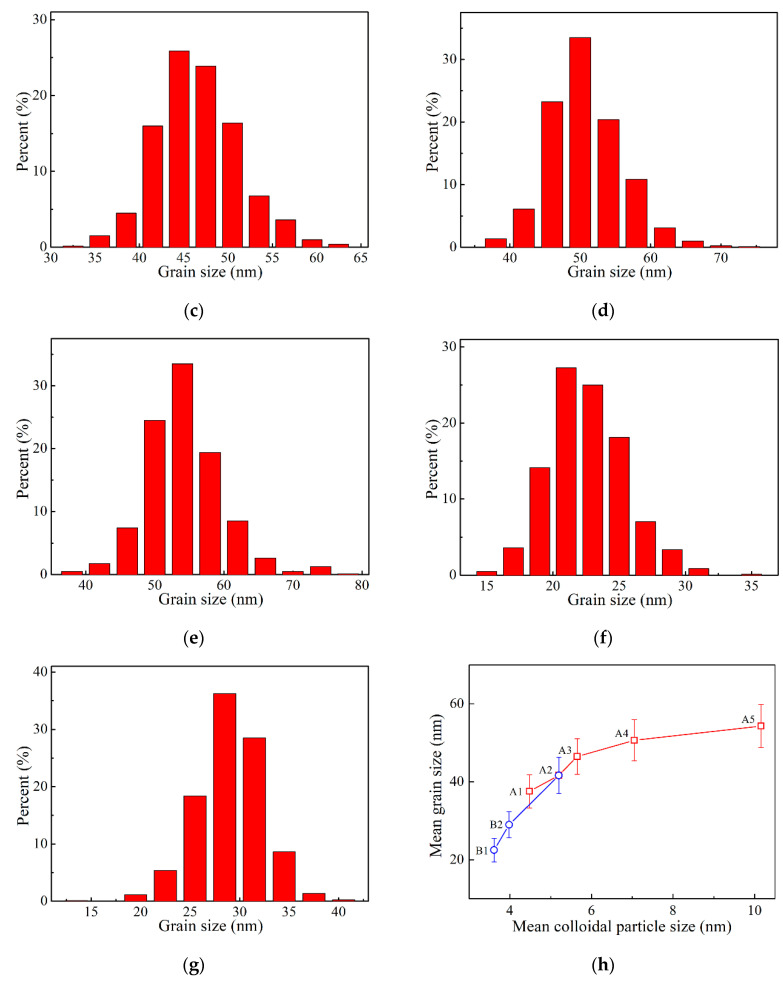
The grain size distribution of alumina fiber: (**a**) Sample A1, (**b**) Sample A2, (**c**) Sample A3, (**d**) Sample A4, (**e**) Sample A5, (**f**) Sample B1 and (**g**) Sample B2. (**h**) The relationship between mean grain size and mean colloidal particle size.

The distribution of fiber grain size is shown in Figure 9. It can be seen that the grain size of each kind of fiber follows the normal distribution. From sample A1 to A5, the peak value increases from 37.5 to 54.0 nm. The sample B1 and B2 exhibit finer grains, which distribute in the range of 14–35 nm and 12–42 nm, respectively. The relationship between the fiber grain size and the iron colloidal particle size is shown in Figure 9h. It was revealed that the maximum mean grain size of fiber (sample A5) was only 54.3 nm. When the mean colloidal particle size decreased from 10.16 to 5.65 nm, the mean grain size also gradually declined from 54.3 to 46.5 nm. With the further decrease of mean colloidal particle size from 5.65 to 3.61 nm, the mean grain size quickly decreased to 22.5 nm. Generally, the grain size of alumina fiber is equal or larger than 100 nm. In this study, the addition of iron sol with small colloidal particle size contributed to forming alumina fiber with nano grains.

**Figure 10:**
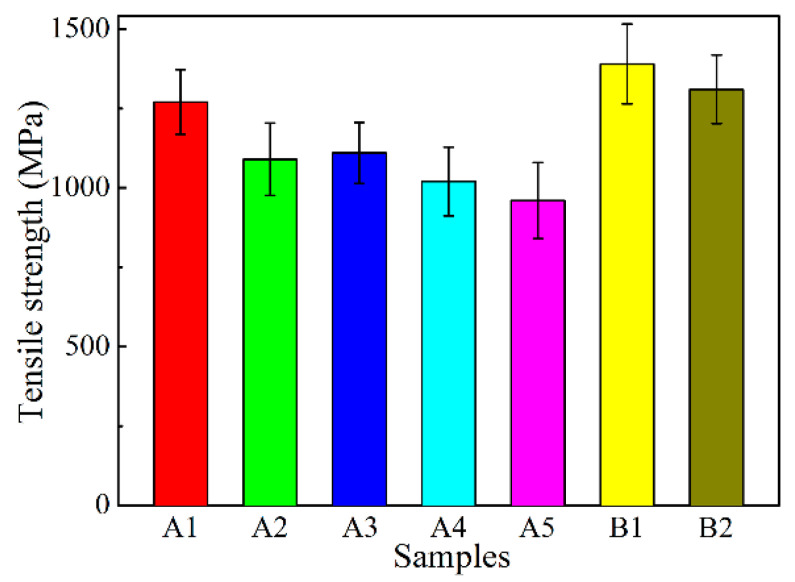
Tensile strength of alumina fibers.

Figure 10 shows the tensile strength of alumina fibers. The sample B1 exhibits the highest tensile strength, up to ~1400 MPa, which is related to the smallest grain size. From sample A1 to A5, the tensile grain size decreases, corresponding to the increase of the grain size. Thus, decreasing fiber grain size contributes to higher tensile strength being acquired.

The formation of α-Al_2_O_3_ includes the nucleation and grain growth processes. Based on the above results, all the iron sols can be employed as nucleating agents to promote the formation of α-Al_2_O_3_. In the nucleation process, the nucleation density of α-Al_2_O_3_ is proportional to the number of iron colloidal particles added in unit volume. When the additional amount is consistent, the reduction of colloidal particle size is effective to increase the colloidal particle number and the nucleation density. Thus, decreasing the colloidal particle size contributes to forming more α-Al_2_O_3_ grains in smaller size. In theory, the initial grain size of α-Al_2_O_3_ increases linearly with the colloidal particle size. However, the α-Al_2_O_3_ grain size rises slightly when the colloidal particle size is larger than 5.65 nm. This probably results from the grain growth of α-Al_2_O_3_. As shown in Figure 7, most of α-Al_2_O_3_ can be formed before 3 min, while the phase transformation completes at 5 min. During this process, the initial α-Al_2_O_3_ grains will grow up and merge into lager grains. Since the growth rate decreases with the increase of Al_2_O_3_ grain size, as a result, the grain size of the samples added with large iron colloidal particles tends to be constant.

## 4. Conclusions

In this study, stable iron sols were prepared with NH_4_HCO_3_ and Fe(NO_3_)_3_·9H_2_O. The mean colloidal particle size ranges from 3.61 to 10.16 nm, which increases with the holding temperature and the NH_4_HCO_3_/Fe(NO_3_)_3_·9H_2_O molar ratio. Sintered at 300 °C for 1 h, the iron sol completely transformed into α-Fe_2_O_3_. Adding iron sol to alumina fiber contributes to forming alumina fiber with uniform nano-scale grains. The minimum mean grain size of fiber is only 22.5 nm, when the iron sol with the mean colloidal particle size of 3.61 nm was used as a nucleating agent. With the increase of colloidal particle size, the grain size tends to rise from 22.5 to 54.3 nm. The fiber with the smallest grain size exhibits the highest tensile strength, up to ~1400 MPa.

## Figures and Tables

**Table 1:** Samples prepared in this study.

Fiber Sample	Iron Sol	Iron Sol Preparation Condition	
		Holding Temperature	NH_4_HCO_3_/Fe(NO_3_)_3_·9H_2_O Ratio
A1	T40	40 °C	2.5
A2	T50	50 °C	2.5
A3	T60	60 °C	2.5
A4	T70	70 °C	2.5
A5	T80	80 °C	2.5
B1	K1.5	50 °C	1.5
B2	K2.0	50 °C	2.0
